# Glass bead games: enumeration of possible polytypes based on two stacking vectors and applications to the iron-ore sinter phases SFCA and SFCA-I

**DOI:** 10.1107/S2053273326003505

**Published:** 2026-04-23

**Authors:** Michael Francesco Salzmann, Volker Kahlenberg, Hannes Krüger, Tim Netzer

**Affiliations:** a Institute of Mineralogy and Petrography University of Innsbruck Innrain 52 Innsbruck TyrolA-6020 Austria; b Institute of Mathematics University of Innsbruck Technikerstr. 13 Innsbruck TyrolA-6020 Austria; Brigham Young University, USA

**Keywords:** polytypism, enumeration of polytypes, SFCA, SFCA-I, iron-ore sintering

## Abstract

Polytypes can occur in many inorganic and organic compounds alike. In the most basic scenario, different polytypes of a compound are based on translationally equivalent layers and two equivalent arrangements of adjacent layers. This means there are only two distinct stacking vectors, **t**_1_ and **t**_2_. For this particular situation, we have enumerated the number of principally different polytypes for a given number *m* of layers in the stacking sequence, under the assumption of the validity of four constraints: (i) periodicity, (ii) exchangeability, (iii) reversibility and (iv) simplicity. Different results are obtained when the reversibility constraint is overturned, a factor that depends on the polar or non-polar character of the underlying layers. The results of this theoretical study have been applied to derive the existing polytypes up to *m* = 6 for two different polysomes of the family of so-called silico-ferrites of calcium and aluminium (SFCA and SFCA-I, respectively). These phases are of considerable interest to the steel industry, as they represent the primary constituents of the matrix in iron-ore sinters. Finally, the impact of polytypism on the powder diffraction patterns of the SFCAs was investigated and the influence of stacking disorder on the diffractograms was analysed. The resulting collection of simulated powder diffraction patterns may serve as a reference database for the identification of polytype effects in industrial sinters.

## Abstract 2

General formulas are presented that allow for the enumeration of polytypes based on translationally equivalent layers and two equivalent arrangements of adjacent layers involving distinct possible stacking vectors, **t**_1_ and **t**_2_. The results have been applied to the polytypism among two different polysomes of the family of so-called silico-ferrites of calcium and aluminium (SFCA and SFCA-I, respectively), which are of importance to iron-ore sintering. Simulations of the powder X-ray diffraction patterns for both ordered and disordered stacking sequences of the SFCAs have been conducted and compiled into a collection of reference diffractograms.

## 1. Introduction

Polytypism can be considered as a special case of polymorphism. In the definition given in the *Online Dictionary of Crystallography* maintained by the Commission for Crystallographic Nomenclature of the International Union of Crystallography, ‘an element or compound is polytypic if it occurs in several different structural modifications, each of which may be regarded as built up by stacking of layers of (nearly) identical structure and composition, and if the modifications differ only in their stacking sequences’. It is based on a suggestion put forth by Guinier *et al.* (1984 ▸). Although this definition can certainly be seen as extremely helpful in understanding the concept of polytypism, there have also been repeated critical comments in the past. One issue of concern was the absence of restrictions in the definition on the sequence and stacking mode of the layers. An excellent summary of the discussion of the various objections and proposed additions has been given by Ďurović (1997 ▸) and Christiansen *et al.* (1999 ▸), for example.

Polytypism as a phenomenon was actually described as early as the 1910s. Indeed, Baumhauer (1912 ▸, 1915 ▸) reported three different types of silicon carbide single crystals using optical goniometry. In the meantime, the number of identified SiC polytypes has risen to approximately 250 (Cheung, 2006 ▸). Since the early days of Baumhauer’s studies, polytypism has been observed in a large number of minerals and mineral groups as well as in synthetic inorganic phases. Natural examples include wollastonite (Dornberger-Schiff *et al.*, 1955 ▸; Ohashi, 1984 ▸), brochantite (Cocco & Mazzi, 1959 ▸; Merlino *et al.*, 2003 ▸), tobermorite (Merlino *et al.*, 1999 ▸), paratobermorite (Pekov *et al.*, 2022 ▸; Kahlenberg & Merlino, 2025 ▸), micas (Nespolo *et al.*, 1997 ▸) and sulfosalts (Moëlo *et al.*, 2008 ▸; Cook *et al.*, 2019 ▸), to name but a few. Synthetic compounds showing polytypism encompass a vast array of chemical compositions. Some examples are CdJ_2_ (Pałosz, 1982 ▸), GaSe (Terhell & van der Sleuten, 1976 ▸), γ-Hg_3_S_2_Cl_2_ (Ďurovič, 1968 ▸), KCa_3_Te_5_­O_12_Cl_3_ (Larvor *et al.*, 2018 ▸), Ca_2.99_Mg_2.67_Fe_15.35_Al_4.56_Si_0.43_O_36_ [also known as SFCA-III (Kahlenberg *et al.*, 2019 ▸)] and RbSbO_3_ (Rotter & Stöger, 2020 ▸). Although the majority of compounds in which polytypism has been observed so far are inorganic in nature, this phenomenon has also been described for a number of organic phases (Timofeeva *et al.*, 2000 ▸; Duggirala *et al.*, 2009 ▸; Britton *et al.*, 2012 ▸; Fröschl *et al.*, 2025 ▸). Comprehensive reviews of polytypic materials have been given by Ďurović & Weiss (1986 ▸), Zvyagin (1988 ▸) and recently Aksenov *et al.* (2023 ▸).

It is important to note that all the previously mentioned examples of polytypic structures, as well as the compounds of interest in this investigation, belong to the group of order–disorder (OD) compounds. The fundamental concepts of OD theory are rooted in the research of Dornberger-Schiff and colleagues (Dornberger-Schiff, 1956 ▸; Dornberger-Schiff, 1964 ▸; Dornberger–Schiff & Fichtner, 1972 ▸). There are several excellent reviews on the theoretical foundations and applications of OD concepts (see, for example, Dornberger-Schiff, 1979 ▸; Merlino, 1997 ▸; Nespolo, 2026 ▸). For that reason, we will just briefly mention some aspects that will facilitate understanding of the content of this paper.

At the core of the OD theory is the use of layers that adhere to three distinct rules. These rules, known as the vicinity condition, delineate the *geometric* equivalence of layer pairs (Dornberger-Schiff, 1979 ▸). Notably, two objects are considered geometrically equivalent if they are congruent. Congruence means that they can be mapped onto each other by an isometry, including translations, rotations and roto-inversions, as well as their combinations. The OD structures, which are built on the same symmetry principle, belong to the same family, also known as the OD groupoid family. A special two-line symbol is used to describe the symmetry of the whole family, providing information on (i) the symmetry of an individual layer (λ-symmetry) and (ii) the symmetry elements mapping a layer onto a neighbouring one (σ-symmetry). Notably, the λ-symmetries conform to one of the 80 layer groups (Grell *et al.*, 1988 ▸). With regard to the diffraction pattern of OD compounds, two types of reflections can be distinguished. The family reflections represent the Fourier transform of the so-called family structure. The family structure encompasses all possible positions of OD layers that are superimposed with equal probability. In a single-crystal diffraction experiment, these peaks are invariably sharp, even in completely disordered crystals, and are consistent across all polytypes within the family. Non-family or characteristic reflections are specific to a particular polytype. These are sharp only in ordered polytypes. Otherwise, they appear as more or less diffuse streaks. Finally, non-space-group absences that do not fit the rules of standard three-dimensional space groups can occur, indicating the presence of a local symmetry operation (Ďurovič, 1999 ▸; Nespolo, 2026 ▸).

**Figure 1:**
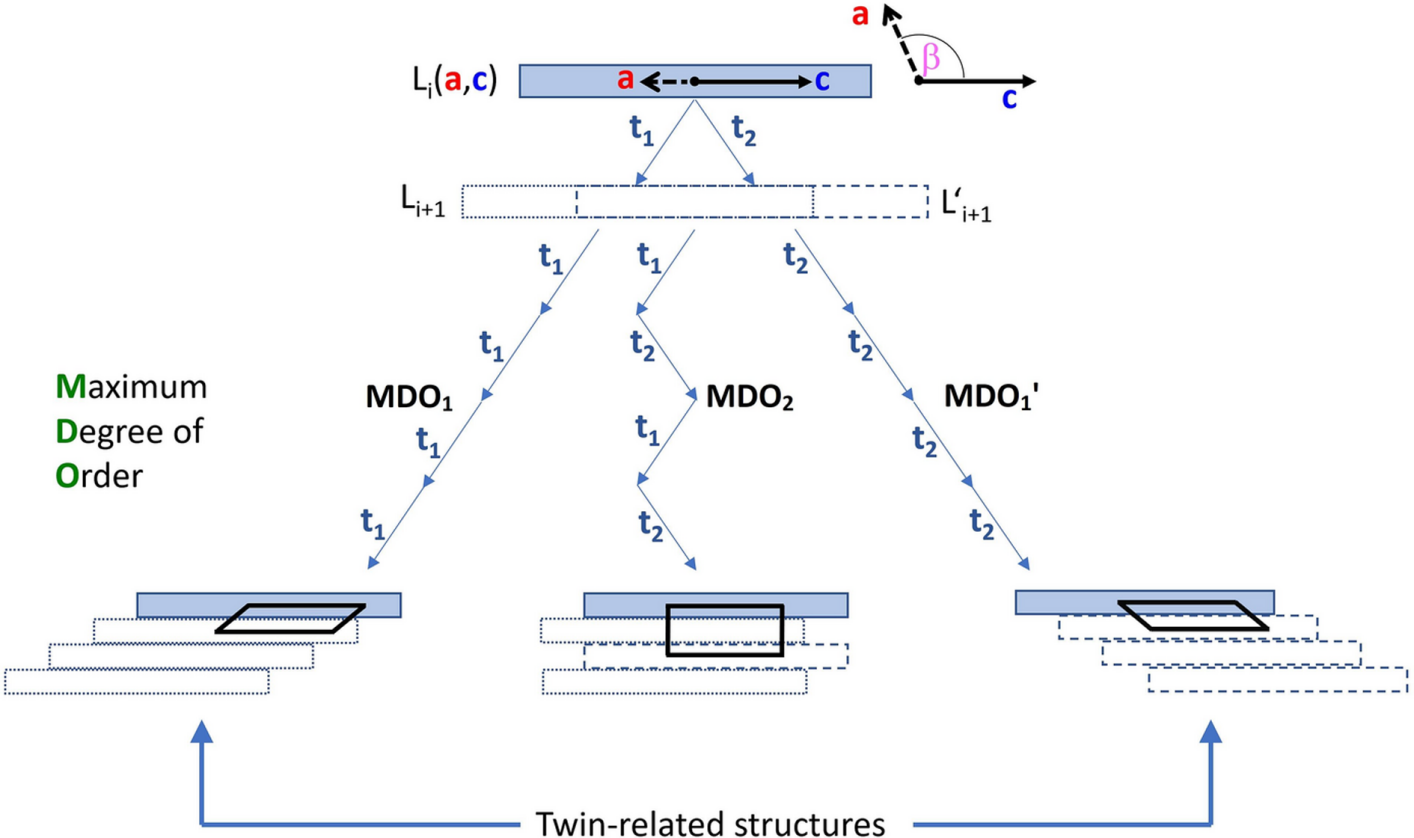
Schematic representation of the simplest periodic stacking sequences (*maximum degree of order structures*) of layers based on the application of two stacking vectors 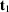 and 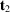: MDO_1_ (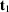) and MDO_2_ (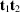). For the sake of clarity, the two basis vectors **a** and **c** defining the periodic layers in two dimensions are shown in projections parallel and perpendicular to the abstract layers. Adapted from Merlino (1997 ▸).

In the simplest case, polytypism in OD structures is based on *translationally* equivalent layers and *two* equivalent arrangements of adjacent layers described by *two* possible vectors, designated as 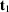 and 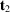. This scenario can be regarded as a special case of geometrical equivalence. The family of wollastonite structures (Merlino, 1990 ▸) is a prominent example of such a situation. In Fig. 1 ▸, the solid rectangle at the top represents a sketch of an abstract two-dimensional periodic layer 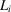, the unit mesh of which is defined by two translation vectors, **a** and **c**. The dashed and dotted lines indicate two potential possibilities for generating a new layer 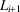 from its predecessor 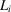. The recurrent application of the shift vectors allows the generation of a plethora of three-dimensional crystal structures by translation. In considering only periodic solutions, one could, for instance, apply solely 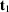 and disregard 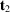. The resulting arrangement of the layers is illustrated on the left of Fig. 1 ▸. Alternatively, only 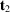 could be employed (see the right part of Fig. 1 ▸). As 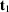 and 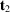 result in equivalent layer pairs, a comparison of the two resulting stacking sequences reveals that they are twins and do not represent fundamentally different structures. By applying 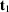 and 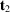 in a strictly alternating fashion, a new sequence is obtained that differs from the first two examples and whose unit cell has increased by a factor of two. The resulting new space group has double the multiplicity and, therefore, the asymmetric unit in both polytypes has the same size. The two cases presented illustrate the simplest and most straightforward stacking sequences, which are also designated as *maximum degree of order structures* (MDOs) (Ďurović, 1997 ▸; Merlino, 1997 ▸).

It is evident that a question of considerable interest is that of the number of polytypes that can be formed for a given number, *m*, of layers in a periodic stacking sequence. Indeed, this question has already been addressed in the literature (Iglesias, 1981 ▸; Mardix, 1990 ▸; Fröschl *et al.*, 2025 ▸). In the majority of cases, the corresponding derivations were based on the stacking of layers assuming packings of equal spheres. On the other hand, the recently published paper by Fröschl *et al.* (2025 ▸) used an organic polytype for the study.

Following the presentation of the results of our more theoretical investigations, we will proceed to apply them in order to derive the possible polytypes up to *m* = 6 for two different polysomes of the family of so-called silico-ferrites of calcium and aluminium (SFCA and SFCA-I, respectively). These phases are of considerable interest to the steel industry, as they represent the primary constituents of the matrix or ‘bonding phase’ in iron-ore sinters (Scarlett *et al.*, 2004 ▸). The concept of incorporating sequences with values for *m* greater than two or three stems from the observation of polytypes with five layers in the stackings of the mineral sapphirine, as shown by transmission electron microscopy studies (Christy & Putnis, 1988 ▸). Notably, sapphirine, with an idealized composition of (Mg,Al)_8_(Al,Si)_6_O_20_, is isostructural with SFCA (Merlino & Zvyagin, 1998 ▸; Zvyagin & Merlino, 2003 ▸).

Finally, we will examine the impact of polytypism on the powder diffraction patterns of the SFCAs and investigate the influence of stacking disorder on the diffractograms. The simulated patterns will facilitate the identification of polytype effects in industrial sinters.

## 2. Counting the numbers of polytypes

**Figure 2:**
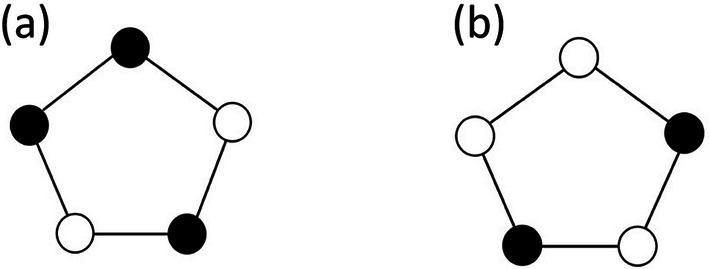
(*a*) Representation of the five-layer polytype 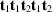 as a cyclic *bbwbw* necklace. (*b*) The exchanged *wwbwb* necklace corresponds to the twin orientation 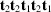.

This section presents the formulas for calculating the number of different possibilities to form polytypes when assembling identical layers. As will be outlined, the symmetry of the layer has an impact on the results. It should be recalled that the objective is to stack *m* layers on top of each other to create a sequence that is then repeated periodically. Notably, we will focus on periodic stacks of *translationally* equivalent layers and two equivalent arrangements of adjacent layers. Each layer in the stack can be shifted in two directions 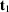 or 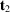 with respect to the previous one, resulting in a total of 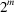 distinct configurations. In the case of 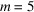, one of the 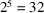 possibilities is 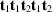, for example. In other words, we are examining all possible strings of glass beads of two different colours, with the colours serving to indicate the specific shift vector that has been applied. If we assign the value *b* (black) for 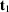 and *w* (white) to 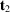, the aforementioned configuration is represented by the string *bbwbw*. The situation is complicated by the fact that not all of these strings are to be considered different. Indeed, two strings are deemed equivalent if they fulfil one of the following constraints:

*Periodicity*. If a configuration is permuted cyclically, that is to say, if the top layer of our stacking sequence is moved to the bottom (also performed repeatedly), then it is considered to be equivalent to the initial one. It is therefore more convenient to think of the beads as being arranged in a cycle, *i.e.* we consider closed necklaces with black and white glass beads. It is evident that the rotation of a necklace does not result in the formation of a new necklace. In this context, the aforementioned example can be visualized as shown in Fig. 2 ▸(*a*).

*Exchangeability*. If the shift vectors 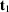 and 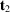 are interchanged at each level of stacking, the resulting structure becomes a *twin* of the initial one, which we wish to consider equivalent. In the context of the necklace configurations, a necklace that is derived from another through the alteration of the colour of each glass bead shall not be regarded as a distinct entity. To illustrate, the necklace shown in Fig. 2 ▸(*b*) is equivalent to the example of Fig. 2 ▸(*a*).

*Reversibility*. If a configuration in a stack is flipped, *i.e.* the layers are considered from bottom to top instead of from top to bottom, the resulting configuration is considered to be equivalent to the initial one. In terms of necklaces, it means that the necklace can be turned over on the table. It is noteworthy that the validity of this constraint depends on the symmetry of the layers that are stacked. This point will be revisited at the end of this section.

*Simplicity*. If a necklace is derived from the repetition of a shorter necklace pattern, it is not necessary to include it in the calculations, as the pattern has already been accounted for as a shorter necklace. For example, the all-black necklace is only to be considered a necklace of length 1, rather than a longer necklace. Similarly, a necklace of length 6 comprising alternating black and white glass beads is not to be considered a necklace of length 6, as it has been already counted as a necklace of length 2.

In accordance with the above-mentioned constraints, there exist precisely three non-equivalent necklaces of length 5, which can be demonstrated through a straightforward argument: 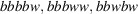. We are now interested in the number of different non-equivalent necklaces, for each length *m*, which fulfil the aforementioned four constraints. The general formula relating *m* with the number of different necklaces is rather involved, and has been reported by Iglesias (1981 ▸). The resulting number sequence is equivalent to the entry A000046 contained in the *On-line Encyclo­pedia of Integer Sequences* (OEIS Foundation Inc., 2025 ▸).

For the case of a prime number *m* we can give a more direct formula, using Pólya’s enumeration theorem:

Theorem 1 If 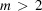 is a prime number, then there exist precisely
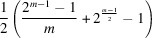
non-equivalent necklaces.

Proof We first consider all 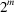 possible strings of black and white beads of length *m*. *Periodicity* and *reversibility* mean that the dihedral group 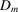 acts on this set and we are interested in the number of different orbits of this action. This can be computed by Pólya’s famous enumeration theorem (Redfield, 1927 ▸; Pólya, 1937 ▸) as
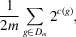
where 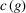 denotes the number of cycles of the element *g* when acting on the strings. Now 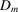 consists of *m* rotations 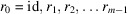 and *m* reflections 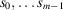. The identity has precisely *m* cycles, while all other rotations have only one cycle (this uses the fact that *m* is prime). Every reflection has precisely one fixed point, once more, given that *m* is prime, and thus precisely 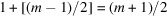 cycles. When this is inserted into Pólya’s formula, the following result is obtained:
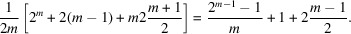

Now we also have to include the *exchangeability* condition. Since 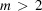 is prime, a string can never have the same number of black and white beads. We can therefore focus on those strings with more black than white beads, and simply divide the above number by 2. Finally, *simplicity* is automatically fulfilled, *i.e.* a necklace cannot have a smaller repeating pattern, again since *m* is a prime. The only necklace we have to exclude is the all-black necklace. Therefore, if the aforementioned formula is divided by two and then subtracted by one, the asserted formula will be obtained.

□

For 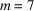 for instance, the expression has a value of 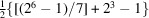, which equals 8, corresponding to the number of principally different polytypes.

When the *reversibility* constraint is bypassed, a slightly different result is obtained. Sequences such as *bbbwwbw* and *bbbwbww* now represent different polytypes. Prior to presenting the most general result, we restrict our consideration to the case where *m* is a prime number. For this case, we can provide a relatively elementary proof once again.

Theorem 2 If 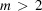 is a prime number, then there exist precisely
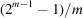
non-equivalent necklaces.

Proof It should first be noted that, given that *m* is not divisible by 2, it is not possible for a necklace to have the same number of black and white glass beads. Thus, by restricting our consideration to necklaces with a greater number of black beads than white, which constitute precisely half of all possible necklaces, or 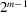 in number, we have ensured the exchangeability condition. Second, given that *m* is a prime number, the only necklace constructed from smaller repeating patterns is the entirely black one, which is not included in the calculations. The remaining 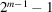 necklaces are then subjected to a cyclic rotation action. It can be demonstrated that a necklace will only repeat itself after a full rotation, since otherwise it would exhibit a smaller repeating pattern, which has already been excluded. Therefore, *m* of the remaining necklaces are obtained via rotation, which leaves precisely
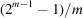
non-equivalent necklaces.

□

The result in its most general form is stated in the following theorem. Once more, the proof is quite technical and involved. It can be found in the work of Fine (1958 ▸). This time, the resulting number sequence is equivalent to the entry A000048 contained in the *On-line Encyclo­pedia of Integer Sequences* (OEIS Foundation Inc., 2025 ▸). It is noteworthy that, in his publication, Fine discussed several abstract problems in number theory, but also highlighted that one of them can be graphically visualized by finding the number of principally different necklaces that can be formed from two-coloured beads considering the aforementioned three side conditions.

**Figure 3:**
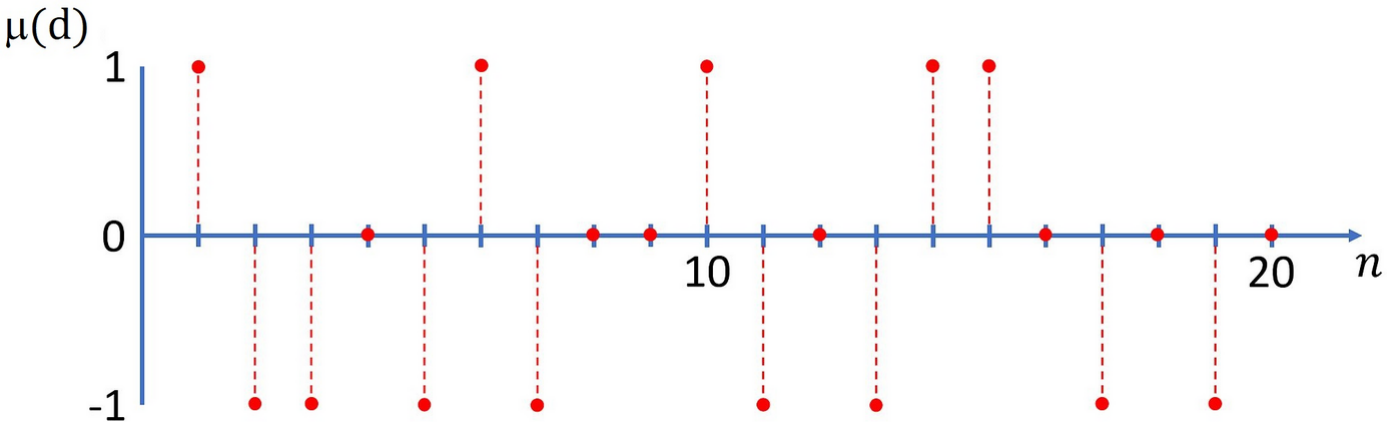
Graphical representation of the distribution of the values of the Möbius function μ(*d*) for the first 20 integers *d*.

Theorem 3 For any 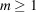 the number of non-equivalent necklaces of length *m* is precisely
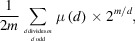
where μ denotes the Möbius function, a well known function in number theory (Hardy & Wright, 1979 ▸) that is defined for natural numbers and can assume only values in the set 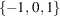. Indeed, 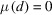 if *d* is not square-free, that is, it contains at least one prime factor with a power of at least 2. If *d* is a square-free integer, then 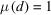 if *d* is a product of an *even* number of different primes, and 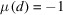 if *d* is a product of an *odd* number of different primes. For example, 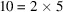 is square-free, while 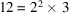 is not. For the sake of clarity, a graphical representation of the values of the Möbius function for the first 20 positive integers is presented in Fig. 3 ▸. The summation in the aforementioned expression is over all odd divisors of *m*. For 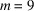 for example, the sum has a value of 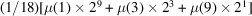 = 28.

As mentioned above, the two different results presented in this section depend on the validity of the *reversibility* condition and, consequently, on the symmetry of the layers that are stacked. Reversibility should be fulfilled when the layers are non-polar, that is, their symmetry belongs to one of the 63 non-polar layer groups. In the case that the layers belong to one of the remaining 17 polar layer groups, reversibility is overturned. We conclude with a table displaying the number of principally different necklaces (number of polytypes) for the first 15 values of *m* for the sequences A000046 and A000048 (see Table 1 ▸). It is noteworthy that both sequences are identical up to *m* = 6.

## 3. Applications to the SFCA family of compounds

### 3.1. General comments on the polysomatic series of the SFCA phases

The members of the SFCA family form a homologous or polysomatic series whose chemical composition can be written as 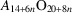, where *A* represents different di-, tri- or tetravalent cations of the elements Ca, Al, Si, Fe and Mg (Zvyagin & Merlino, 2003 ▸; Webster *et al.*, 2012 ▸; Nicol *et al.*, 2018 ▸). Depending on the value of *n*, different family members are distinguished, which are designated by Roman numbers: SFCA (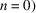, SFCA-I (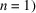 and SFCA-III (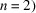 (Hamilton *et al.*, 1989 ▸; Mumme *et al.*, 1998 ▸; Liles *et al.*, 2016 ▸; Kahlenberg *et al.*, 2019 ▸; Kahlenberg *et al.*, 2021*a* ▸). An SFCA-II phase (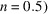 has also been described which is an intermediate between SFCA and SFCA-I (Mumme, 2003 ▸; Kahlenberg *et al.*, 2021*b* ▸). The series can be formed from a combination of two types of layers (slabs) which can be imagined as being cut from the well known spinel (S) and pyroxene (P) crystal structures. A detailed analysis of the relationships between the family members including concepts of OD theory has been given by Merlino & Zvyagin (1998 ▸), Zvyagin & Merlino (2003 ▸) and Kahlenberg *et al.* (2019 ▸).

**Figure 4:**
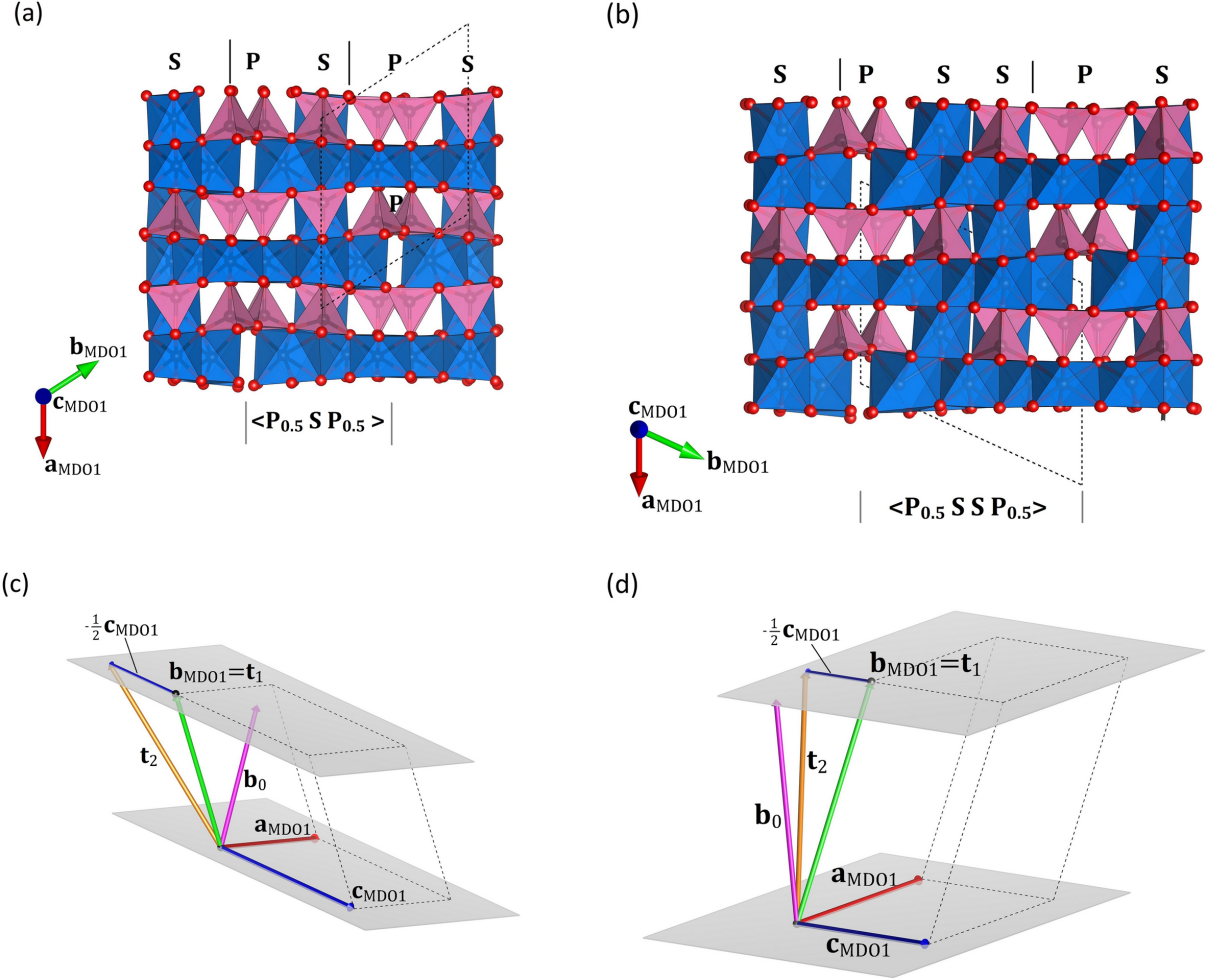
(*a*) and (*b*) illustrate a sequence of 〈PS〉 and 〈PSS〉 modules in SFCA and SFCA-I, respectively, in projections parallel to 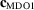, that is, parallel to the tetrahedral pyroxene chains. Tetrahedra and octahedra are presented in pink and blue, respectively. Small red spheres denote oxygen atoms. The boundaries of the 〈PS〉 and 〈PSS〉 modules, as well as the OD layers, which are all parallel to (010), are indicated with small solid black and grey lines, respectively. The outlines of the unit cells of the MDO1 structures are shown with black stippled lines. For the sake of clarity, the vector relationships between the translation (shift) vectors 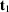 and 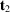, as well as the unit-cell vectors 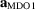, 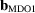 and 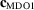 have been shown separately in (*c*) and (*d*) for SFCA and SFCA-I, respectively. The two grey planes indicate the boundaries of a single OD layer. Please note that both 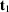 and 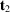 are situated within the plane defined by 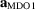 and 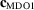. 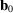 corresponds to the vector perpendicular to the OD layers (see text).

In summary, the members of the polysomatic series differ in the ratio of the S and P slabs in the stacking sequences: while SFCA is based on a 〈PS〉 sequence containing one pyroxene and one spinel slab, the other modules of the representatives are characterized by the following sequences: SFCA-I: 〈PSS〉, SFCA-III: 〈PSSS〉, SFCA-II: 〈PSPSS〉. As Zvyagin & Merlino (2003 ▸) have demonstrated, when employing the framework of OD theory for description, it is more convenient to denote the OD layers as 〈P_½_SP_½_〉, 〈P_½_SSP_½_〉 and 〈P_½_SSSP_½_〉, respectively. In other words, the boundary planes have been shifted with respect to 〈PS〉, 〈PSS〉 and 〈PSSS〉 to describe the inherent symmetry more easily. For the sake of clarity, Figs. 4 ▸(*a*) and 4 ▸(*b*) show the individual P and S slabs, the 〈PS〉 and 〈PSS〉 modules and the OD layers that can be defined in SFCA and SFCA-I, respectively.

According to Zvyagin & Merlino (2003 ▸) only two types of OD groupoid families have to be considered in the poly­somatic series of SFCA compounds:
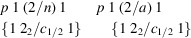
The left symbol describes the symmetry characteristics of polysome 〈PS〉 (or 〈P_½_SP_½_〉), while the right symbol refers to the symmetry features of 〈PSS〉 (or 〈P_½_SSP_½_〉).

As mentioned above, the first line of each symbol describes the layer group of a single OD layer, while the second line contains the information about the σ-operations. Both symbols for the layer groups are based on a setting resulting in a monoclinic angle β of about 110°. In both cases, the layers are non-polar. The vector 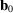 is along the direction normal to the *a*–*c* plane with the magnitude 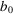 corresponding to the thickness of a single OD layer (Merlino & Zvyagin, 1998 ▸; Zvyagin & Merlino, 2003 ▸). Notably, one of the symmetry elements that relates adjacent layers is a glide plane perpendicular to 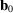 with translational components 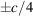 labelled as 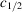 and 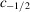. The second σ-operation is a twofold rotation axis with a translational component corresponding to **b**_0_ and, therefore, denoted 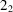.

Following the concepts of OD structures, an adjacent layer pair related through 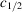 is *geometrically* equivalent to a pair related by 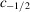. As shown by Zvyagin & Merlino (2003 ▸), in this particular case the 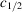 and the 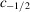 operators correspond to two translations: 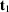 = 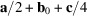 and 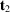 = 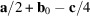. As a result, subsequent layers are *translationally* equivalent. Notably, the magnitudes of 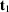 and 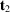 are different.

In the case of SFCA-III, Kahlenberg *et al.* (2019 ▸) have demonstrated that this particular polysome can indeed be present in different polytypes. The structure determination of both polytypes was performed using the same sample that was identified as a so-called allotwin, that is to say, an oriented intergrowth of two distinct polytypes. The resulting triclinic and monoclinic structures were found to correspond to the MDO_1_ (*m* = 1) and MDO_2_ (*m* = 2) variants, which can be obtained from the application of two possible stacking vectors 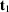 or 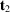 to a single layer-like 〈PSSS〉 module of S and P slabs (see also Fig. 1 ▸). In other words, MDO_1_ is formed from the application of 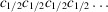 (or the twinned structure 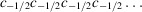) and MDO_2_ by 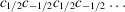. Furthermore, Salzmann *et al.* (2024 ▸) recently synthesized a highly disordered SFCA (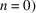 sample and demonstrated that the two simplest polytypes, corresponding to *m* = 1 and *m* = 2 (the latter representing a hypothetical phase), can be derived from this basic or family structure through ordering processes of cations among different possible octahedral and tetrahedral vacancies.

To date, the phenomenon of polytypism in real samples of the SFCA series has only been observed in SFCA-III. However, there is no reason to believe that this phenomenon is exclusive to this particular family member and that it should not occur in other SFCAs as well, which are also based on 〈P〉 and 〈S〉 slabs. It should be noted that the industrial sinter conditions are far away from equilibrium, particularly in comparison with laboratory experiments, which typically last for 24 or 48 h before final quenching. In a typical sinter furnace, the gas-burner-induced flame front, with temperatures of about 1300°C, is sucked downwards through the several-decimetres-thick sinter bed in only a few minutes, producing large temperature gradients and variations in oxygen fugacity. These rather harsh industrial conditions should facilitate the formation of distinct polytypes within the main sinter phases, namely SFCA and SFCA-I. So far, however, no detailed polytype-related investigations on SFCAs retrieved from a sinter bed have been performed.

The individual OD layers involved in the formation of the potential polytypes in SFCA and SFCA-I are *translationally* equivalent and non-polar. Therefore, their numbers for a given number *m* of layers in the stack can be predicted using the A000046 sequence listed in Table 1 ▸.

### 3.2. Procedure to derive the potential ordered polytypes of SFCA and SFCA-I

In order to derive the potential polytypes of SFCA and SFCA-I we decided to consider the cases with up to 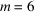 layers in the stacking sequence. The starting points were two high-quality crystal structure refinements for the simplest MDO_1_ (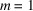) polytypes with the following chemical compositions: Ca_2.24_Fe_8.68_Al_1.36_Mg_0.15_Si_0.76_O_20_ (for SFCA, Liles *et al.*, 2016 ▸) and Ca_3.36_Fe_12.71_Al_3.94_O_28_ (for SFCA-I, Kahlenberg *et al.*, 2021*a* ▸). Both polytypes conform to the triclinic space group 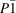 and contain exactly one 〈PS〉 or 〈PSS〉 module within the unit cell.

According to the published structure data, most of the tetrahedral (T) and octahedral (M) positions show mixed-site populations involving two or even more cation species. Moreover, vacancies on the cation sites may occur as well. In order to simplify the partially complex cation distribution patterns, the relevant cation positions in both phases were occupied with a single atom species that matched the average electron density, as determined from diffraction-based site population analysis, as closely as possible. As an illustration, an M site with a population of 7% Mg, 2% Ca and 91% Fe corresponds to a total of 24.9 electrons and was therefore modelled as a ‘virtual’ Mn atom (atomic number 25). Furthermore, the crystallographic data presented in the two aforementioned papers pertain to the reduced triclinic cells. For our polytype analysis, however, we finally decided to follow the aforementioned suggestion given by Zvyagin & Merlino (2003 ▸) for SFCAs that (i) the lattice vectors defining the layer-like structural units are labelled **a** and **c**, and (ii) that both vectors are selected in such a manner that they include an angle of approximately 110°. Consequently, a transformation of the unit cell was necessary and the resulting new sets of lattice parameters can be found in Tables 2 ▸ and 3 ▸. With respect to this new cell, the two vectors reflecting the translation symmetry of the layers correspond to 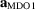 and 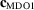.

To avoid any wrong presumptions regarding the symmetry that may have an impact on the polytype derivation, it was further deemed necessary to reduce the symmetry of the MDO_1_ structures from 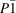 to 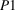. Therefore, we finally obtained representations of the individual modules for each of the two SFCA polysomes under investigation, based on a total of 16 M sites, 12 T sites and 40 oxygen positions (〈PS〉, SFCA) and 24 M sites, 16 T sites and 56 oxygen position (〈PSS〉, SFCA-I).

For the next step of our polytype construction, it is of crucial importance that the two potential stacking vectors 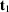 and 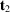 can be consistently expressed by the unit-cell vectors of the simplest MDO_1_ polytypes of SFCA and SFCA-I. The relevant two vector relationships are as follows: 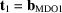 and 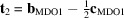. For the sake of clarity, Figs. 4 ▸(*c*) and 4 ▸(*d*) present the different vectors involved in the description of 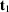 and 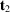.

**Figure 5:**
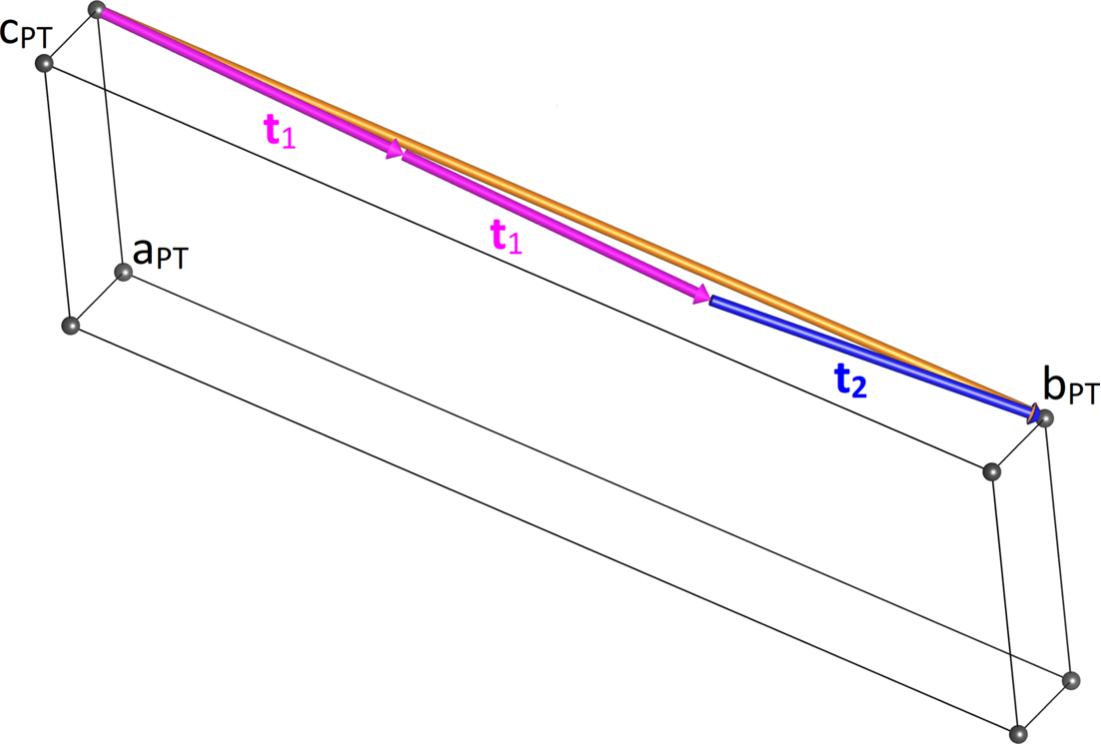
Derivation of the unit cell of the 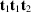 polytype of SFCA-I. The unit-cell vector 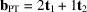 indicates the stacking direction. 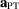 and 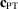 define a single 〈PSS〉 layer.

Therefore, it is now possible to predict the unit-cell vectors of all polytypes (PT). Two of them correspond to the layer-defining vectors, that is, 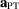 = 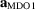 and 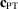 = 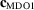. The missing vector 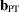 points along the stacking direction and can be calculated independently for each particular sequence. As an example, for the specific 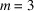 sequence 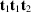 the following matrix equation is obtained (see also Fig. 5 ▸):
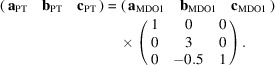
The resulting values for the corresponding SFCA-I family member, for example, are as follows: 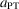 = 10.4061, 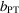 = 35.3759, 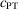 = 10.5783 Å, α_PT_ = 94.34°, β_PT_ = 110.17°, γ_PT_ = 65.41°. With respect to this new coordinate system the stacking vectors have the following components: 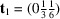 and 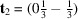.

**Figure 6:**
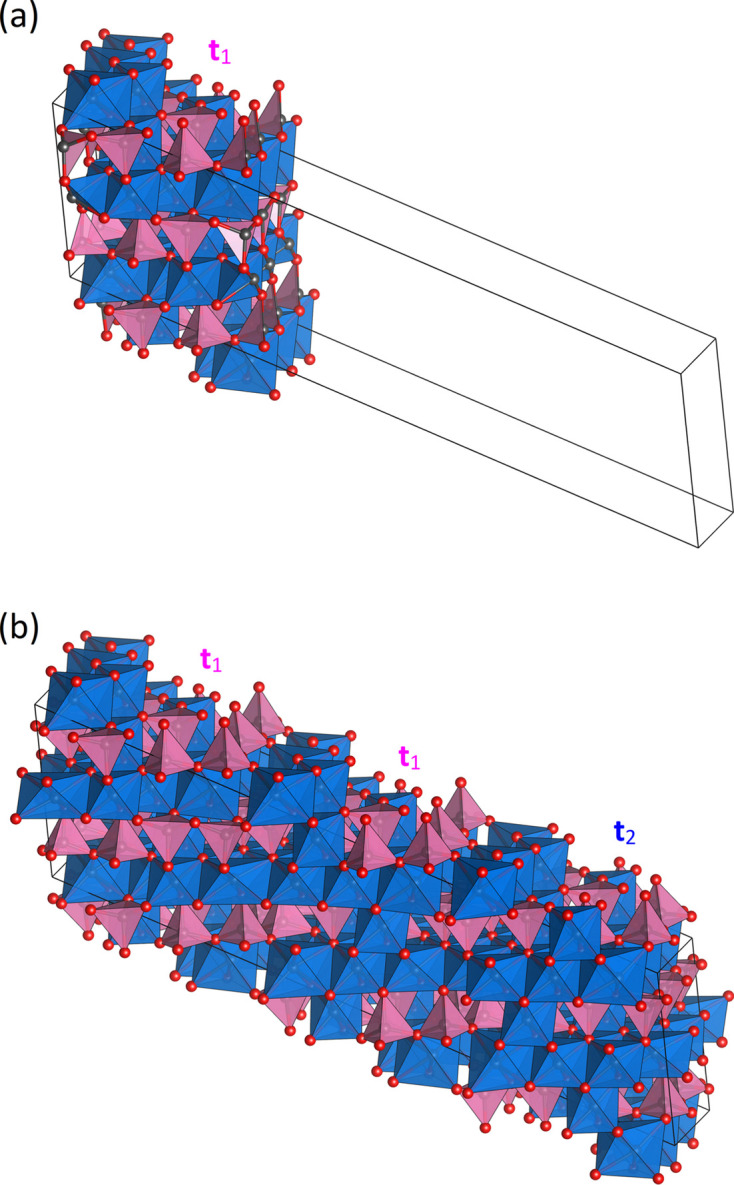
(*a*) Insertion of a single 〈PSS〉 module into the unit cell of the 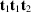 polytype of SFCA-I. (*b*) Resulting crystal structure of the 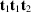 polytype after the application of all three relevant stacking vectors.

Subsequently, the crystal structures of all polytypes can be derived by inserting the first single 〈PS〉 or 〈PSS〉 module into each of the new cells [see Fig. 6 ▸(*a*)] and applying the specific stacking sequences to generate the missing modules, thereby filling the cells completely [see Fig. 6 ▸(*b*) for the 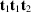 example in SFCA-I]. Assuming *identical* layers, each position 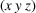 in module 1 of the 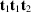 sequence is equivalent to a position with the same site scattering density in 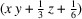 in module 2 and 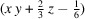 in module 3.

Following this protocol, it is now possible to obtain the complete structural characterization of all polytypes in space group 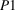. Finally, we investigated whether the resulting structures had been described in an unnecessarily low space-group symmetry. In order to test for potential higher symmetries, we employed a consecutive combination of the program *ADDSYM* implemented in the *PLATON* software suite (Spek, 2003 ▸) and the program *PSEUDO* (Kroumova *et al.*, 2001 ▸), a module of the *Bilbao Crystallographic Server* (Aroyo *et al.*, 2006 ▸). The results of the symmetry search process for the SFCA and SFCA-I based polytypes are also summarized in Tables 2 ▸ and 3 ▸. It should be noted that we finally decided to describe the resulting monoclinic space-group types in a standard setting, cell choice 1 (*P*2_1_/*c*, *Pc*). For the SFCA polytype, this results in a monoclinic angle of β ≃ 124°. Should the angle between the two layer-defining vectors be set to β ≃ 110° (see above), then cell choice 2 of space group No. 14 should be selected. This will change the space-group symbols to *P*2_1_/*n* and *Pn*, respectively. Furthermore, we would like to reiterate that the results presented so far are specific to the scenario where the individual layers within the sequence are identical.

In particular, the program *PSEUDO* has the additional benefit of facilitating a detailed analysis of those sites that are equivalent under the symmetry operations of the potentially higher symmetrical space groups. This process may be termed ‘Wyckoff merging’ (following the well known term ‘Wyckoff splitting’ used for the reverse operation upon symmetry reduction). Depending on the established higher symmetry, either two or four sites could be merged. Notably, the symmetry search was performed with a focus on the atomic coordinates, that is, differences in the individual site populations of the M and T sites were initially neglected. In order to generate the resulting CIF files for all polytypes, which are provided as supporting information, it was finally decided to retain the previously generated ‘virtual’ atomic species on the different cation positions. This was to ensure that the electron densities of these sites match the average of the merged real distributions as closely as possible (see above).

## 4. Simulation and interpretation of the powder diffraction patterns

### 4.1. Preliminary theoretical considerations

The objective of the approach outlined in Section 3.2 was to develop structure models that facilitate a realistic simulation of the resulting powder diffraction patterns of the polytypes. It is notable that our assumption of stacking *identical* layers, however, has direct consequences in the form of non-space-group absences (see the *Introduction*). This aspect will be explored in more detail using the 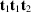 polytype as an example. In light of the aforementioned discussion of the equivalent positions in the three relevant layers, the structure factor for this particular stacking sequence, as described in 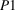, can be written as follows:
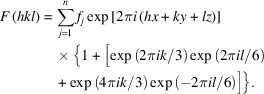
The summation is over all *n* atoms belonging to module 1. It is evident that 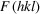 is equal to zero if the term inside the square brackets, which depends only on *k* and *l*, is equal to 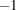. A detailed analysis of this expression resulted in the following systematic absences:

(i) If 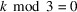: reflections are absent if *l* is congruent to 2 or 4 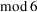.

(ii) If 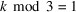: reflections are absent if *l* is congruent to 0 or 2 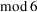.

(iii) If 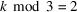: reflections are absent if *l* is congruent to 0 or 4 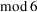.

Notably, two integers *f* and *e* are *congruent modulo p* if there is an integer *q* such that *f* − *e* = *qp*.

In particular, the first two possible observable reflections of the powder pattern of the triclinic 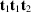 structure, specifically 010 [case (ii)] and 020 [case (iii)], are forbidden, whereas 030 [case (i)] is observable. In fact, all polytypes based on larger values of *m* layers in the sequence show a similar behaviour: only those reflections with 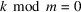 can be observed for the 0*k*0 class (if the polytypes are referred to the triclinic cells used before analysing potential higher symmetries). This result is, to some extent, unfavourable, as it is precisely these low-angle reflections that emerge with increasing values of *m* at progressively lower diffraction angles. Consequently, they would be ideally suited to differentiating between polytypes with varying numbers of layers or modules in the stacking sequence. Generally speaking, a more detailed and critical analysis of the peaks in the low 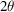 regions is needed to tell the polytypes apart.

**Figure 7:**
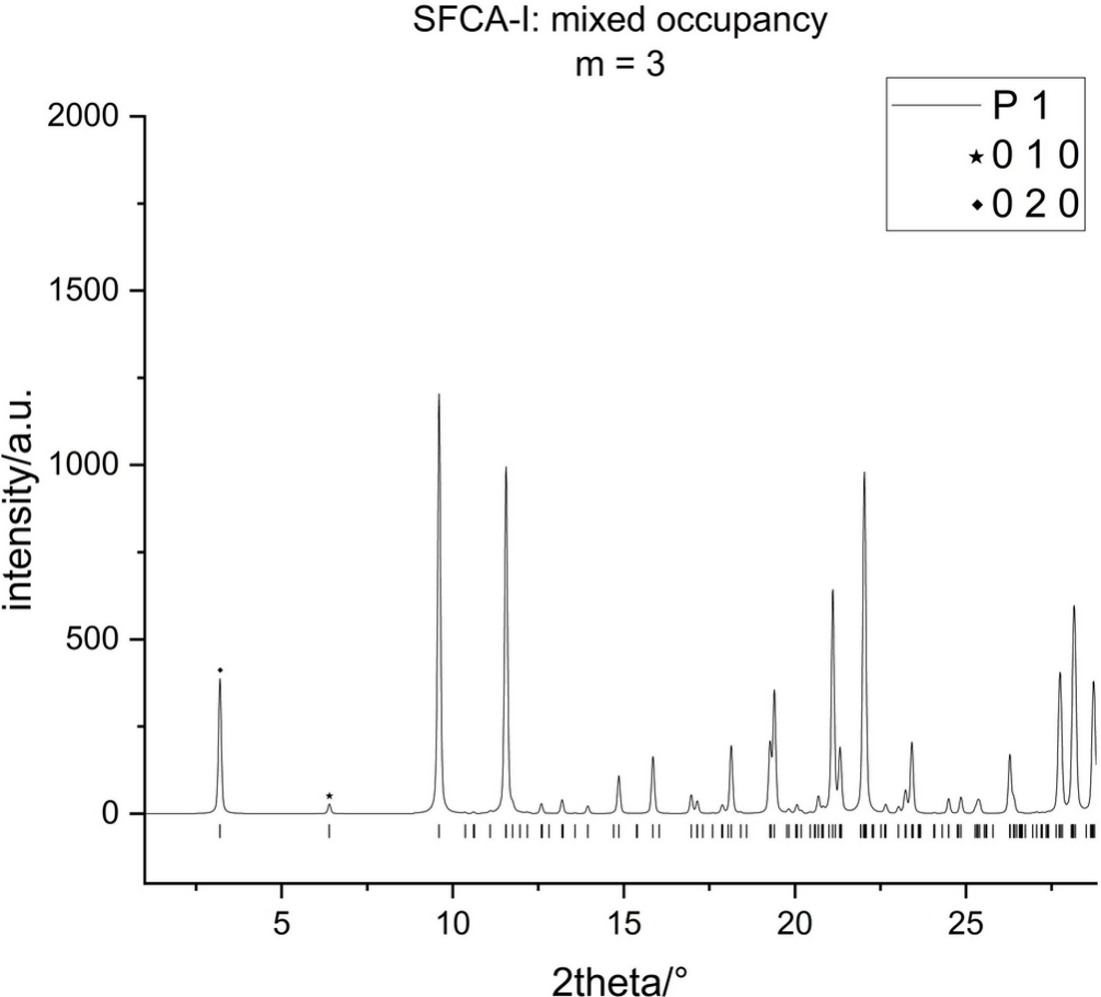
Powder X-ray diffraction pattern for the 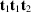 polytype of SFCA-I simulated in space group 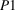 for Co *K*α_1_ radiation under the assumption that the electron densities of the cation species in the three principal modules within the unit cell differ by ±1% (see text). The critical newly appearing low-angle peaks 010 and 020 have been marked with an asterisk.

However, the situation changes if the premise of the identity of all layers within the sequence is abandoned. To provide an example, we simulated a powder pattern for the 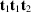 polytype of SFCA-I in space group 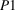, where the scattering densities of the M and T sites belonging to the second and third modules differed by minus 1% (layer II) and plus 1% (layer III) from the corresponding values for the first layer. As expected, the critical low-angle peaks (010, 020) now appear and could be used for the identification of the polytypes with different values of *m* (see Fig. 7 ▸). More information on the technical details of the calculation of the powder patterns will be provided in the following section.

### 4.2. Distinguishing the ordered polytypes of SFCA and SFCA-I in PXRD: practical aspects

**Figure 8:**
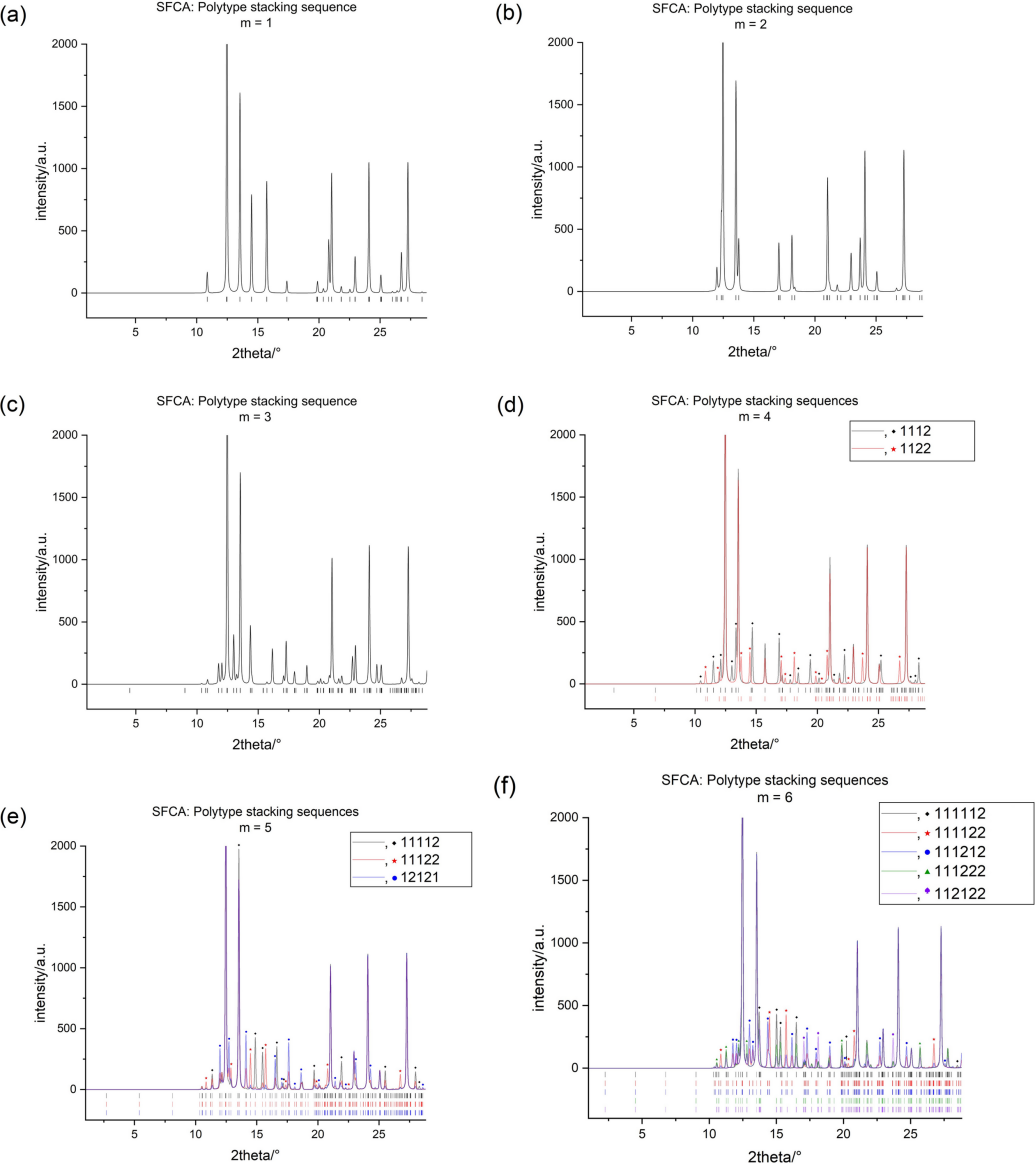
Compilation of the simulated powder X-ray diffraction patterns in the low 2θ range (Co *K*α_1_ radiation) for the potential ordered SFCA polytypes up to *m* = 6.

The analysis was performed using the program *Jana2006* (Petříček *et al.*, 2014 ▸) by simulating the powder X-ray diffraction (PXRD) patterns of the polytypes under uniform conditions using the previously derived structure models (see Section 3.2). For modelling of the peaks, pseudo-Voigt functions were employed with values for peak widths, shape and asymmetry that are typical for the standard configuration of the Stadi-MP diffractometer located at the Mineralogical Institute in Innsbruck when operated in Bragg–Brentano geometry with a sealed cobalt tube and *K*α_1_ radiation generated from a Ge(111) primary beam monochromator. The step size was set to 0.01° 2θ. Intensities within 12 times the full width at half-maximum of a peak were considered to contribute to the central reflection. The diffraction angle range was restricted to the interval between 1° and 29° 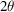, thereby ensuring a sufficient separation of all reflections belonging to a particular polytype. The resulting set of powder patterns may serve as a reference database for the identification of a particular polytype (see Figs. 8 ▸ and 9 ▸).

It is evident that powder diffraction patterns of well ordered polytypes adhere to the same general principles as observed in any other crystalline compound. While the peak positions are defined by the unit-cell parameters, the distribution of the different atoms and atomic species inside the cell governs the distribution of weak and strong reflections. Furthermore, the number of peaks observed may be subject to further reduction due to systematic absences related to the corresponding space-group symmetries. As outlined in the previous section, the SFCAs show additional systematic non-space-group extinction rules, the identification of which may necessitate a thorough analysis of the diffraction patterns. Finally, it is a well established fact that polytypes belonging to the same modular family typically exhibit similar diffraction patterns (Merlino, 1997 ▸).

As summarized in Tables 2 ▸ and 3 ▸, for both SFCA and SFCA-I, only one possible polytype is observed for stacking sequences involving *m* = 1, 2 and 3. For *m* = 4, the diffraction patterns of the two existing polytypes for both SFCA and SFCA-I are easily distinguishable. Due to differences in space-group symmetry and unit-cell parameters, each polytype shows a sufficiently large number of unique reflections (see Figs. 8 ▸ and 9 ▸). Conversely, for 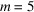, the three possible polytypes share the same triclinic space group 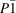 and exhibit identical cell parameters, *i.e.* there are no unique peaks. It is possible to differentiate between them, but this requires a careful analysis of the distribution of peak intensities (see Figs. 8 ▸ and 9 ▸). For the five simulated polytypes of *m* = 6, three of them (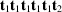, 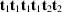 and 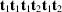) adopt space group 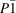. In this set of three, the 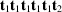 polytype shows different unit-cell parameters compared with the other two polytypes, leading to different reflection positions. Because the metrics of the unit cells of 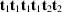 and 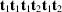 are identical, a differentiation can only be achieved through a comparison of their peak intensities. In contrast, the remaining two monoclinic polytypes (space groups *P*2_1_/*c* and *Pc*) exhibit different systematic space-group-related systematic absences. The 2_1_ screw axis along [010] affects the (0*k*0) peaks with *k* ≠ 2*n* in *P*2_1_/*c*, but not in *Pc*. Unfortunately, this difference is only of limited value, because of the non-space-group absences discussed in Section 4.1.

Following on from these more general comments, which highlight some characteristic common features and issues related to identifying stacking sequences that are valid for the polytypes of both polysomes, we attempted to define reflections that could be used for the purpose of differentiation. To achieve this objective, we sought to identify sets of three diffraction peaks for each of the various polytypes that can be formed for a given *m* value. Ideally, these reflections would be sufficiently intense and would not overlap with those from other stacking variants. As the latter condition could not be met in all cases, care was taken to guarantee that the potentially overlapping peaks of the other sequences had low intensities at the corresponding 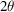 values. This ensured that the observation of such a peak is still decisive for identification. Another issue to consider is that some of these reflections – given the resolution function of the selected diffractometer – correspond to a superposition of family and characteristic reflections, which cannot be resolved experimentally (see above). The results of the analysis are summarized in Tables 4 ▸ and 5 ▸, with peaks involving contributions from family reflections being marked by an asterisk. Furthermore, the given cells (see Sections 4.3.1 and 4.3.2) of the family structures for SFCA and SFCA-I can be used for rapid identification of those compounds in powder diffraction patterns, as they will match regardless of the presence of ordered polytypes or disorder. In a subsequent step, the identification of signs of diffuse scattering or specific patterns of superstructure reflections can be attempted.

### 4.3. Simulation of the diffraction patterns of the disordered structures

To obtain reference powder diffraction patterns of disordered materials intermediate between the fully ordered and simplest polytypes 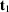 (= MDO_1_) and 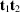 (= MDO_2_) of SFCA and SFCA-I, simulations were performed using the software *discus* (version 6.19.00) (Proffen & Neder, 1997 ▸; Proffen & Neder, 1999 ▸; Neder & Proffen, 2025 ▸). As starting models, the same symmetry-reduced 〈PS〉 or 〈PSS〉 layers (for SFCA and SFCA-I, respectively) were used as those utilized in Section 3.2 to derive the ordered polytypes.

Taking into account the two possible stacking vectors given in Section 3.2 (namely 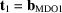 and 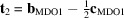), a faulted model can be set up using a Markov chain growth disorder model, which is defined by a so-called right stochastic matrix *M*, a square matrix consisting of non-negative real numbers, with each row adding up to 1. The matrix contains the probabilities between adjacent stacking vector layers (*e.g.*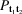 is the probability that a vector 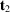 follows after a vector 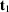),
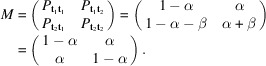
In general, the growth parameters α and β are related to the elements of the stochastic matrix as shown above (for details, see *e.g.* Welberry, 2004 ▸; Neder & Proffen, 2008 ▸). The growth parameter α represents the probability of a change in the stacking vector, whereas β denotes the correlation between neighbouring vectors. Assuming equal amounts of 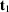 and 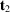 vectors, this simplifies as shown above (β = 1 − 2α).

The stacking-fault mode of *discus* was used to build the model, and thereafter diffraction patterns were calculated using the ‘stack’ option of the Fourier mode, where the diffraction of the full model crystal is calculated as the product of the Fourier transform of the list of origins (of the simulated stack of layers) and the Fourier transform of one layer. Because of computational restrictions, a single simulation run was performed for 10^3^ layers, and larger models were mimicked by averaging the diffraction patterns of 10^2^ simulations. As a result, each simulation contains the statistics of 10^5^ layers. Powder patterns were derived by complete integration of the reciprocal space. Pseudo-Voigt profile functions and wavelength (Co *K*α_1_) as described in Section 4.2 were utilized.

Using this procedure, powder patterns for ordered and disordered models with order parameters α = 0.0, 0.25, 0.50, 0.75 and 1.0 were derived. The limiting cases α = 0.0 and 1.0 obviously simulate the fully ordered polytypes 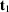 (= MDO_1_) and 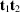 (= MDO_2_), respectively. As a test, a comparison with the diffraction patterns calculated with *Jana2006* from the polytype models shows an almost perfect match. Note that minor intensity differences are caused by symmetry merging and thus averaging of scattering factors.

#### 4.3.1. Disorder in SFCA

**Figure 10:**
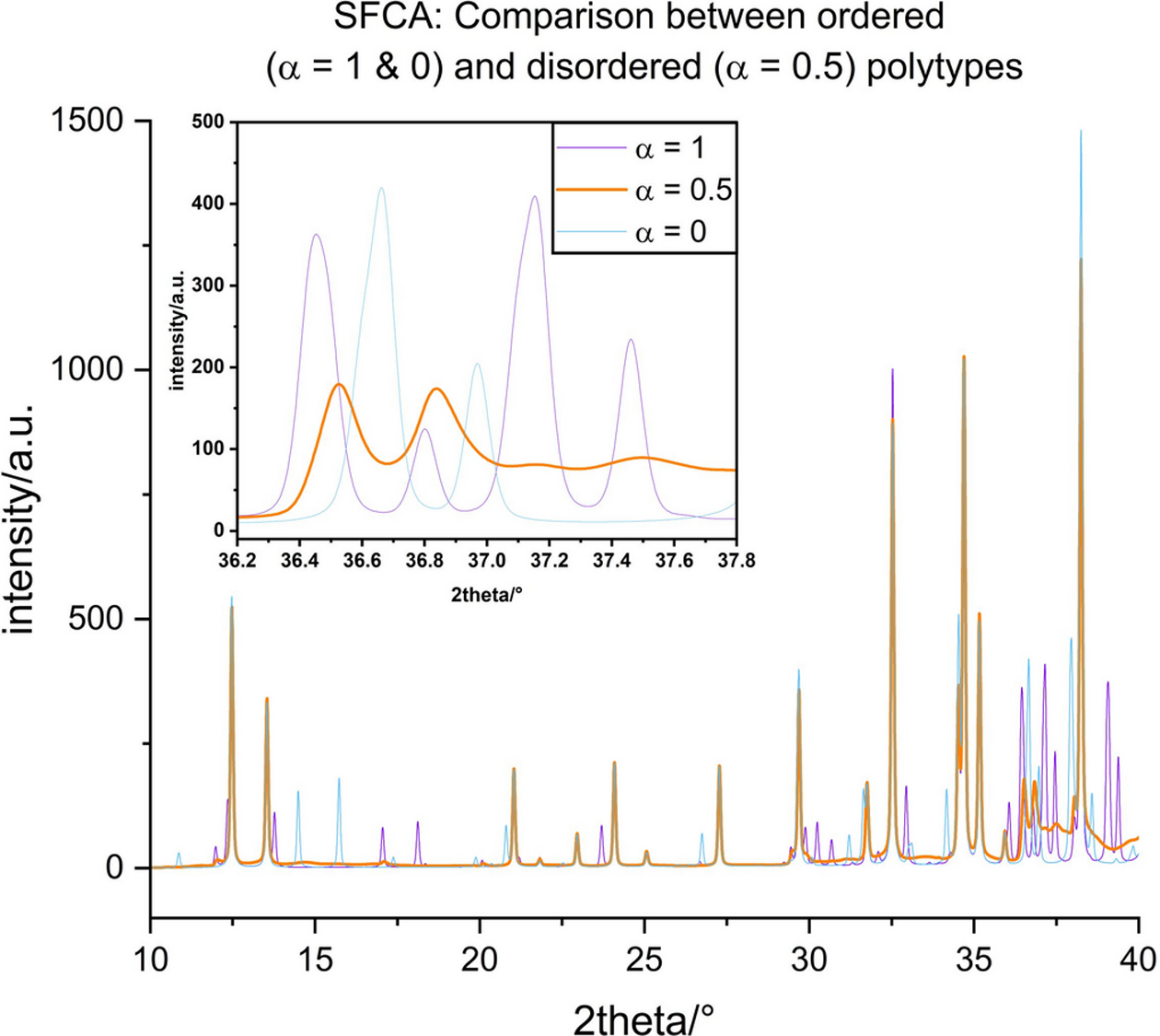
Comparison between the simulated powder diffraction patterns in a small 2θ range of ordered (α = 1 or MDO_2_ and α = 0 or MDO_1_) and completely disordered (α = 0.5) SFCA (see text).

When the layers are *translationally* equivalent, a single diffraction study shows Bragg peaks on rows with family reflections and diffuse scattering corresponding to the structure factor of an individual layer on rows with characteristic reflections. The calculated powder diffraction pattern of the randomly disordered SFCA (α = 0.5) exhibits a significantly different pattern, compared with the ordered MDOs 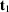 and 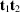 (see Fig. 10 ▸). Obviously, all MDO-related superstructure reflections are missing. Furthermore, diffuse scattering intensity is noticeable in some places, although the maximum intensity is rather low (less than 5% of the strong peaks). It can be assumed that in experimental patterns this feature will be hard to detect. The most obvious differences between the diffraction patterns of ordered and disordered SFCA are visible in the range of 36.2–37.8° 2θ (see inset of Fig. 10 ▸). We found that this pattern can be indexed with a monoclinic *C*-centred cell of *a* = 9.960, *b* = 15.175, *c* = 5.280 Å, β = 100.285°, which is the cell of the family structure, as was also observed recently in a disordered SFC (silico-ferrite of calcium) structure with composition Ca_2.68_Fe_10.32_Si_1.00_O_20_, although given in another setting (monoclinic *I*-centred, Salzmann *et al.*, 2024 ▸).

#### 4.3.2. Disorder in SFCA-I

**Figure 11:**
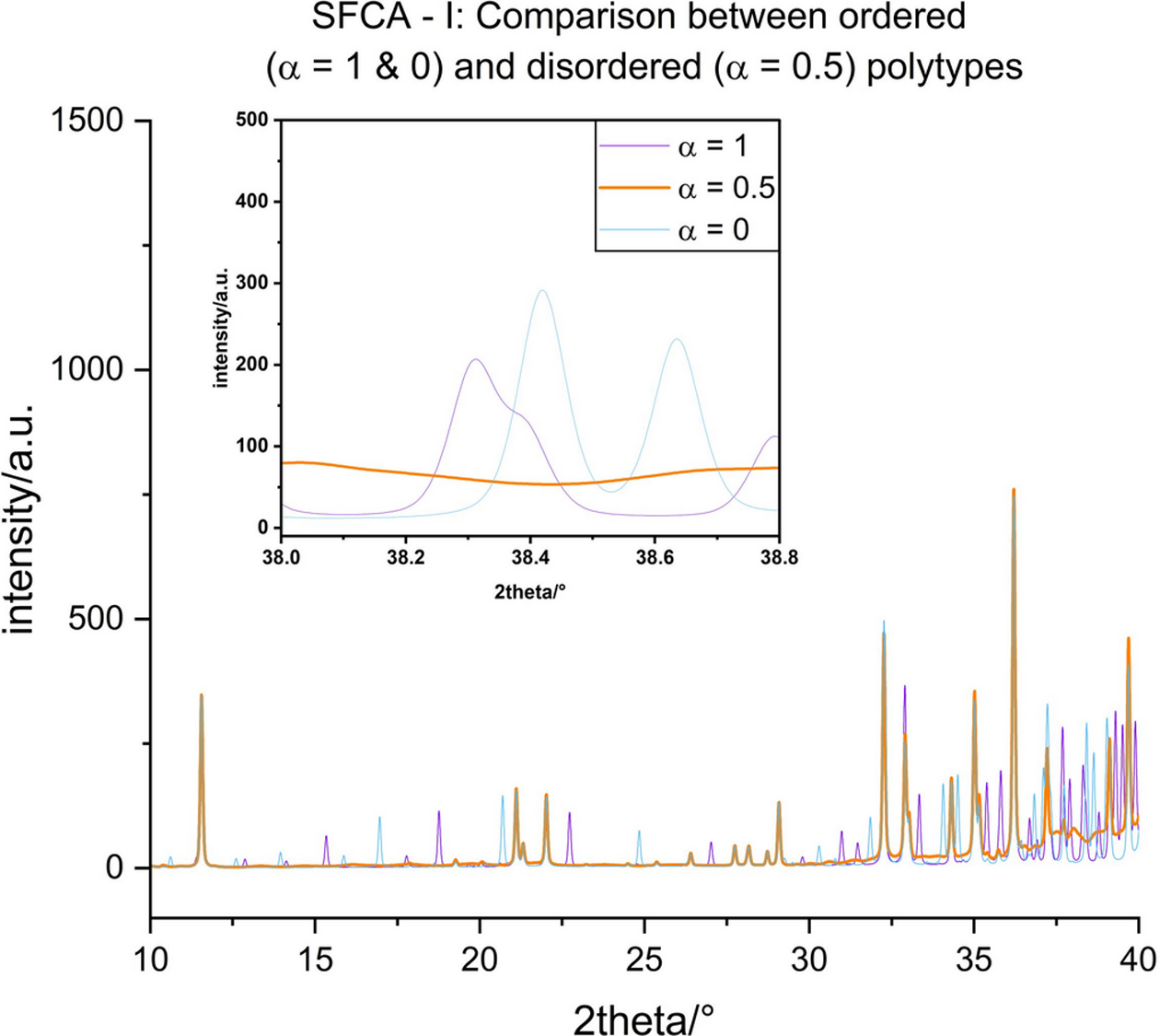
Comparison between the simulated powder diffraction patterns in a small 2θ range of ordered (α = 1 or MDO_2_ and α = 0 or MDO_1_) and completely disordered (α = 0.5) SFCA-I (see text).

The SFCA-I unit cell of a single 〈PSS〉 layer as derived from a synthesized sample labelled SFCA-I-04 (Kahlenberg *et al.*, 2021*a* ▸) (entry 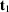 in Table 3 ▸) does not exactly transform to a monoclinic cell for the 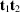 polytype, even though the deviations of the angles α and γ from 90° are very small (see Table 3 ▸, entry 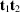). In the case of the simulation with *discus* this leads to visible peak splitting in the powder diffraction patterns of the **t**_1_**t**_2_ polymorph. Therefore, we used a slightly adjusted cell (*a* = 10.4061, *b* = 11.7826, *c* = 10.5783 Å, α = 85.861°, β = 110.17°, γ = 68.642°), which perfectly transforms to a monoclinic cell for 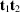. In order to maintain consistency, the same adjusted cell was used to calculate the diffraction pattern of the ordered 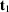 polytype. However, it has to be noted that this simplifies the diffraction pattern of 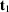, as a monoclinic supercell is present for the adjusted cell parameters. The simulated pattern of disordered (growth parameter α = 0.5) SFCA-I can be indexed with a monoclinic *C*-centred cell of *a* = 9.915, *b* = 21.378, *c* = 5.289 Å, β = 99.88°. Again, this cell should correspond to the metric of the family structure of SFCA-I. The largest deviations due to diffuse scattering can be found between 38.0 and 38.8° 2θ (see the inset of Fig. 11 ▸).

## 5. Concluding remarks

As outlined in Section 2, the equations presented are transferable and can be applied to all polytype problems where *translationally* equivalent layers are stacked on top of each other using two different translation vectors, 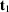 and 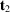. This means that there are two equivalent arrangements of neighbouring layers. Therefore, it is possible to predict the number of principally different polytypes for a given number *m* of layers in the sequence, independent of the structure under investigation. The only factor that requires consideration is whether the layers are polar or non-polar. In the event that the vectors 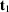 and 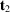 can be related to the vectors defining the simplest polytype (MDO_1_), it is possible to generate not only the unit-cell parameters of all possible more complex polytypes, but also to derive the corresponding crystal structures. The principal procedure has been outlined in some detail using the compounds SFCA and SFCA-I as examples.

A simulation of periodic SFCA and SFCA-I polytypes with *m* = 1 to 6 layers in the stacking sequence was carried out to establish a consistent basis for their identification in powder diffraction data. For values of *m* = 1, 2 and 3, there is only one polytype and a differentiation between them using PXRD is straightforward. Polytypes belonging to *m* = 4 can also be easily distinguished by characteristic non-overlapping reflections resulting from differences in the unit cell and symmetry. In contrast, the polytypes with *m* = 5 and several with *m* = 6 share identical triclinic unit cells, leading to completely overlapping reflection positions. As a result, identification is necessarily based on variations in relative intensities related to their stacking sequences. It is important to note that in some rare cases peaks involving contributions from family reflections had to be included. The combined assessment of reflection positions, extinction rules and intensity distributions provides an applicable framework for identifying individual polytypes in these complex polysomatic systems. Once identified, the polytype models that we derived could also be used as structural input for the subsequent quantitative phase analysis, which is now based on the Rietveld method.

At any rate, the discrimination requires the use of high-resolution, high-quality powder X-ray diffraction data. Data collections at synchrotron light sources would be especially well suited for this purpose. It is evident that such data are not generally available for immediate use. Instead, they require the submission of proposals that are subject to approval, even though new modes of access have been currently implemented and may improve the availability for a broader community (Hinrichsen *et al.*, 2024 ▸). Therefore, the simulations that we provided were deliberately performed using the resolution functions and peak shape parameters of typical diffractograms acquired for highly crystalline samples on a sealed-tube instrument equipped with a primary beam monochromator, yielding monochromatic *K*α_1_ radiation. This configuration represents the ‘best you can get’ under laboratory conditions. Diffraction patterns measured on a standard Bragg–Brentano instrument using *K*α_1_ and *K*α_2_ are not the optimal choice for a polytype analysis, particularly when comparatively short data collection times are employed for data acquisition. Unfortunately, the literature on SFCAs from materials science and metallurgy includes a large number of powder diffraction patterns that are not suitable for achieving the aforementioned objective.

Moreover, it should be noted that even more complicated polytypes among the SFCAs may exist. It is evident that the crystal structure of the aforementioned SFCA-II polysome, characterized by the stacking sequence 〈PSPSS〉, can be considered a combination of SFCA (〈PS〉) and SFCA-I (〈PSS〉) in a 1:1 ratio. However, it is possible that other sequences involving combinations of these two layer types with different ratios, such as 〈PSPSPSS〉 or 〈PSPSSPSS〉, for example, could also occur. A more detailed analysis of these polytypic variants could prove fruitful.

Finally, additional complementary transmission electron microscopy investigations on both laboratory and industrial sinters would be extremely beneficial in identifying potential polytypic stacking patterns among the silico-ferrites of calcium and aluminium.

## Supplementary Material

ZIP file containing the structural information of the ordered polytypes for SFCA in CIF format, used for the simulation of the powder <?print bk [?tjl=100mm]$6#[?tjl]$w>X-ray<?print ek> diffraction patterns. DOI: 10.1107/S2053273326003505/cam5016sup1.zip

ZIP file containing the structural information of the ordered polytypes for SFCA-I in CIF format, used for the simulation of the powder <?print bk [?tjl=100mm]$6#[?tjl]$w>X-ray<?print ek> diffraction patterns. DOI: 10.1107/S2053273326003505/cam5016sup2.zip

## Figures and Tables

**Figure 9:**
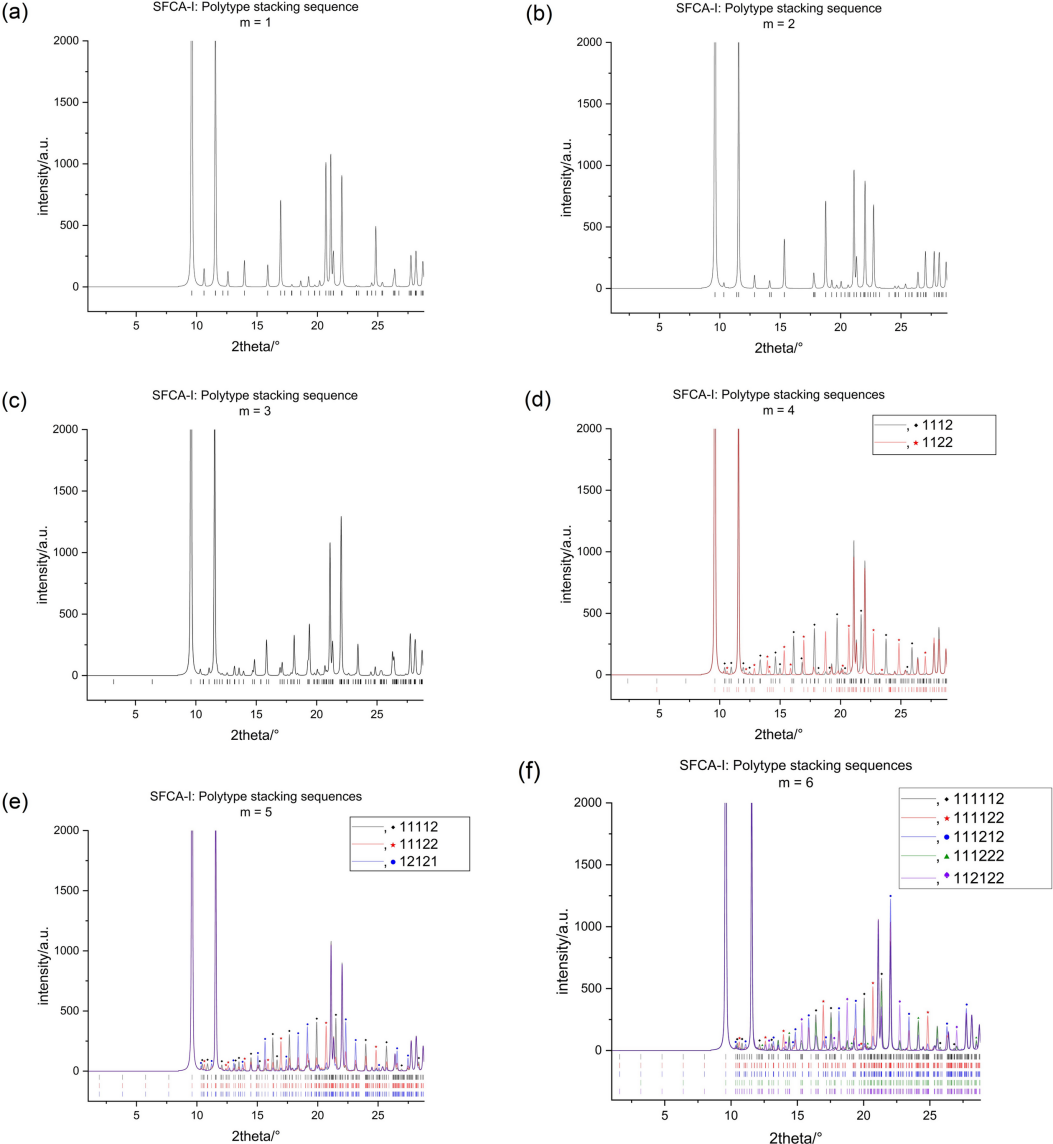
Compilation of the simulated powder X-ray diffraction patterns in the low 2θ range (Co *K*α_1_ radiation) for the potential ordered SFCA-I polytypes up to *m* = 6.

**Table 1:** Number of principally different polytypes (necklaces) for the first 15 values of *m* in the case that the reversibility condition is valid (first line) or not (second line) The resulting sequences correspond to the entries A000046 and A000048 of the *On-line Encyclo­pedia of Integer Sequences* (OEIS Foundation Inc., 2025 ▸). Note: *m* corresponds to the number of layers in the stacking sequence.

*m*	1	2	3	4	5	6	7	8	9	10	11	12	13	14	15
No. of polytypes (A000046)	1	1	1	2	3	5	8	14	21	39	62	112	189	352	607
No. of polytypes (A000048)	1	1	1	2	3	5	9	16	28	51	93	170	315	585	1091

**Table 2:** Summary of the basic crystallographic data of the SFCA polytypes up to six layers (*m* = 6) Columns 2 and 3 list the 3 × 3 transformation matrices for the lattice parameters (given as row vectors) and the resulting magnitudes and angles for the polytypes in *P*1. The first line of column 4 represents the transformation matrices (**P**, **p**) for the atomic coordinates (given as column vectors) for the description in higher symmetry. The matrices **P**^−1^ for transforming the lattice parameters are listed on the second line of column 4. Column 5 summarizes the resulting new lattice parameters. Remark: the vectors **a**_MDO1_ and **c**_MDO1_ are located within a single OD layer (β ≃ 110°). For the monoclinic polytypes, the small deviations of the angles α and γ from their ideal values after transformation were neglected, and both angles can be considered to be 90°.

Stacking sequence of the SFCA polytypes	Transformation matrix with respect to **a**_MDO1_, **b**_MDO1_, **c**_MDO1_	Transformed cell	Transformation matrices (**P**, **p**) and **P**^−1^ to the monoclinic or the reduced cell (in case of a resulting triclinic symmetry)	Monoclinic or reduced triclinic cell	Resulting higher space group
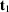 (MDO_1_)	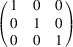	*a*_MDO1_ = 10.4074 Å	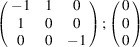	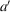 = 9.0738 Å	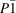
		*b*_MDO1_ = 9.0738 Å		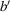 = 10.0480 Å	
		*c*_MDO1_ = 10.5611 Å		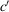 = 10.5611 Å	
		α_MDO1_ = 95.64°	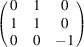	α′ = 64.06°	
		β_MDO1_ = 109.67°		β′ = 84.36°	
		γ_MDO1_ = 118.35°		γ′ = 65.72°	
		*V* = 785.39 Å^3^		*V*′ = 785.39 Å^3^	
					
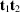 (MDO_2_)	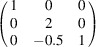	*a* = 10.4074 Å	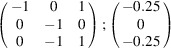	*a*′ = 10.4074 Å	*P*2_1_/*c*
		*b* = 19.3921 Å		*b*′ = 15.1781 Å	
		*c* = 10.5611 Å		*c*′ = 12.0771 Å	
		α = 111.36°	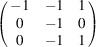	α′ = 90.03°	
		β = 109.67°		β′ = 124.57°	
		γ = 110.65°		γ′ = 89.95°	
		*V* = 1570.77 Å^3^		*V*′ = 1570.93 Å^3^	
					
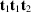	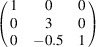	*a* = 10.4074 Å	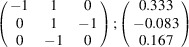	*a*′ = 10.4074 Å	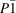
		*b* = 28.2337 Å		*b*′ = 10.5611 Å	
		*c* = 10.5611 Å		*c*′ = 23.2990 Å	
		α = 106.37°	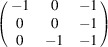	α′ = 92.21°	
		β = 109.67°		β′ = 100.63°	
		γ = 113.26°		γ′ = 109.67°	
		*V* = 2356.16 Å^3^		*V*′ = 2356.10 Å^3^	
					
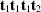	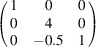	*a* = 10.4074 Å	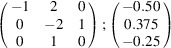	*a*′ = 10.4074 Å	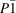
		*b* = 37.1873 Å		*b*′ = 10.5611 Å	
		*c* = 10.5611 Å		*c*′ = 30.8091 Å	
		α = 103.76°	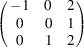	α′ = 80.15°	
		β = 109.67°		β′ = 86.74°	
		γ = 114.56°		γ′ = 70.33°	
		*V* = 3141.55 Å^3^		*V*′ = 3141.73 Å^3^	
					
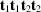	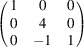	*a* = 10.4074 Å	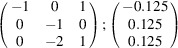	*a*′ = 10.4074 Å	*P*2_1_/*c*
		*b* = 38.7843 Å		*b*′ = 30.3562 Å	
		*c* = 10.5611 Å		*c*′ = 12.0771 Å	
		α = 111.36°	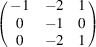	α′ = 90.03°	
		β = 109.67°		β′ = 124.57°	
		γ = 110.65°		γ′ = 89.95°	
		*V* = 3141.55 Å^3^		*V*′ = 3141.86 Å^3^	
					
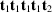	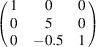	*a* = 10.4074 Å	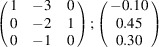	*a*′ = 10.4074 Å	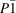
		*b* = 46.1879 Å		*b*′ = 10.5611 Å	
		*c* = 10.5611 Å		*c*′ = 38.2701 Å	
		α = 102.17°	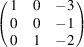	α′ = 91.31°	
		β = 109.67°		β′ = 96.52°	
		γ = 115.34°		γ′ = 109.67°	
		*V* = 3926.93 Å^3^		*V*′ = 3926.94 Å^3^	
					
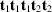	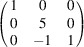	*a* = 10.4074 Å	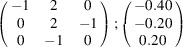	*a*′ = 10.4074 Å	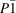
		*b* = 47.5822 Å		*b*′ = 10.5611 Å	
		*c* = 10.5611 Å		*c*′ = 38.2656 Å	
		α = 108.40°	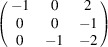	α′ = 91.35°	
		β = 109.67°		β′ = 96.42°	
		γ = 112.21°		γ′ = 109.67°	
		*V* = 3926.93 Å^3^		*V*′ = 3927.17 Å^3^	
					
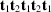	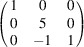	*a* = 10.4074 Å	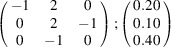	*a*′ = 10.4074 Å	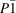
		*b* = 47.5822 Å		*b*′ = 10.5611 Å	
		*c* = 10.5611 Å		*c*′ = 38.2656 Å	
		α = 108.40°	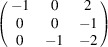	α′ = 91.35°	
		β = 109.67°		β′ = 96.42°	
		γ = 112.21°		γ′ = 109.67°	
		*V* = 3926.93 Å^3^		*V*′ = 3927.17 Å^3^	
					
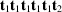	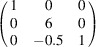	*a* = 10.4074 Å	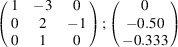	*a*′ = 10.4074 Å	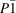
		*b* = 55.2124 Å		*b*′ = 10.5611 Å	
		*c* = 10.5611 Å		*c*′ = 45.5368 Å	
		α = 101.10°	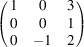	α′ = 89.98°	
		β = 109.67°		β′ = 89.95°	
		γ = 115.85°		γ′ = 70.33°	
		*V* = 4712.32 Å^3^		*V*′ = 4712.50 Å^3^	
					
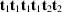	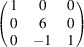	*a* = 10.4074 Å	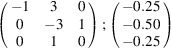	*a*′ = 10.4074 Å	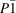
		*b* = 56.4674 Å		*b*′ = 10.5611 Å	
		*c* = 10.5611 Å		*c*′ = 45.8296 Å	
		α = 106.37°	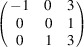	α′ = 83.41°	
		β = 109.67°		β′ = 87.82°	
		γ = 113.26°		γ′ = 70.33°	
		*V* = 4712.32 Å^3^		*V*′ = 4712.02 Å^3^	
					
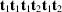	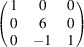	*a* = 10.4074 Å	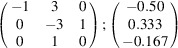	*a*′ = 10.4074 Å	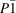
		*b* = 56.4674 Å		*b*′ = 10.5611 Å	
		*c* = 10.5611 Å		*c*′ = 45.8296 Å	
		α = 106.37°	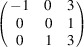	α′ = 83.41°	
		β = 109.67°		β′ = 87.82°	
		γ = 113.26°		γ′ = 70.33°	
		*V* = 4712.32 Å^3^		*V*′ = 4712.02 Å^3^	
					
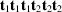	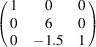	*a* = 10.4074 Å	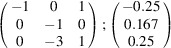	*a*′ = 10.4074 Å	*P*2_1_/*c*
		*b* = 58.1764 Å		*b*′ = 45.5342 Å	
		*c* = 10.5611 Å		*c*′ = 12.0771 Å	
		α = 111.36°	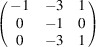	α′ = 90.03°	
		β = 109.67°		β′ = 124.57°	
		γ = 110.65°		γ′ = 89.95°	
		*V* = 4712.32 Å^3^		*V*′ = 4712.78 Å^3^	
					
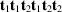	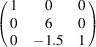	*a* = 10.4074 Å	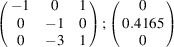	*a*′ = 10.4074 Å	*Pc*
		*b* = 58.1764 Å		*b*′ = 45.5342 Å	
		*c* = 10.5611 Å		*c*′ = 12.0771 Å	
		α = 111.36°	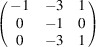	α′ = 90.03°	
		β = 109.67°		β′ = 124.57°	
		γ = 110.65°		γ′ = 89.95°	
		*V* = 4712.32 Å^3^		*V*′ = 4712.32 Å^3^	

**Table 3:** Summary of the basic crystallographic data of the SFCA-I polytypes up to six layers (*m* = 6) Columns 2 and 3 list the 3 × 3 transformation matrices for the lattice parameters (given as row vectors) and the resulting magnitudes and angles for the polytypes in *P*1. The first line of column 4 represents the transformation matrices (**P**, **p**) for the atomic coordinates (given as column vectors) for the description in higher symmetry. The matrices **P**^−1^ for transforming the lattice parameters are listed on the second line of column 4. Column 5 summarizes the resulting new lattice parameters. Remark: the vectors **a**_MDO1_ and **c**_MDO1_ are located within a single OD layer (β ≃ 110°). For the monoclinic polytypes, the small deviations of the angles α and γ from their ideal values after transformation were neglected, and both angles can be considered to be 90°.

Stacking sequence of the SFCA-I polytypes	Transformation matrix with respect to **a**_MDO1_, **b**_MDO1_, **c**_MDO1_	Transformed cell	Transformation matrices (**P**, **p**) and **P**^−1^ to the monoclinic or the reduced cell (in case of a resulting triclinic symmetry)	Monoclinic or reduced triclinic cell	Resulting higher space group
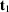 (MDO_1_)	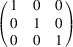	*a*_MDO1_ = 10.4061 Å	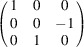	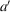 = 10.4061 Å	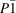
		*b*_MDO1_ = 11.7905 Å		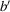 = 10.5783 Å	
		*c*_MDO1_ = 10.5783 Å		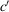 = 11.7905 Å	
		α_MDO1_ = 85.76°	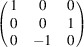	α′ = 94.24°	
		β_MDO1_ = 110.17°		β′ = 111.38°	
		γ_MDO1_ = 68.62°		γ′ = 110.17°	
		*V* = 1104.47 Å^3^		*V*′ = 1104.47 Å^3^	
					
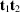 (MDO_2_)	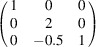	*a* = 10.4061 Å	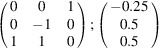	*a*′ = 10.5783 Å	*P*2_1_/*c*
		*b* = 23.7823 Å		*b*′ = 21.3772 Å	
		*c* = 10.5783 Å		*c*′ = 10.4061 Å	
		α = 98.57°	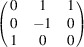	α′ = 89.98°	
		β = 110.17°		β′ = 110.17°	
		γ = 64.01°		γ′ = 89.88°	
		*V* = 2208.93 Å^3^		*V*′ = 2208.86 Å^3^	
					
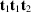	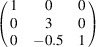	*a* = 10.4061 Å	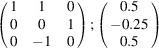	*a*′ = 10.4061 Å	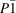
		*b* = 35.3759 Å		*b*′ = 10.5783 Å	
		*c* = 10.5783 Å		*c*′ = 32.4557 Å	
		α = 94.33°	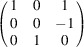	α′ = 91.62°	
		β = 110.17°		β′ = 97.64°	
		γ = 65.41°		γ′ = 110.17°	
		*V* = 3313.40 Å^3^		*V*′ = 3313.26 Å^3^	
					
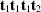	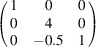	*a* = 10.4061 Å	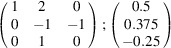	*a*′ = 10.4061 Å	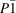
		*b* = 47.0674 Å		*b*′ = 10.5783 Å	
		*c* = 10.5783 Å		*c*′ = 43.0705 Å	
		α = 92.19°	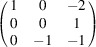	α′ = 83.06°	
		β = 110.17°		β′ = 87.54°	
		γ = 66.17°		γ′ = 69.83°	
		*V* = 4417.86 Å^3^		*V*′ = 4417.83 Å^3^	
					
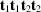	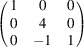	*a* = 10.4061 Å	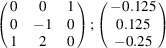	*a*′ = 10.5783 Å	*P*2_1_/*c*
		*b* = 47.5645 Å		*b*′ = 42.7543 Å	
		*c* = 10.5783 Å		*c*′ = 10.4061 Å	
		α = 98.57°	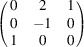	α′ = 90.04°	
		β = 110.17°		β′ = 110.17°	
		γ = 64.01°		γ′ = 90.12°	
		*V* = 4417.86 Å^3^		*V*′ = 4417.71 Å^3^	
					
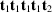	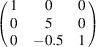	*a* = 10.4061 Å	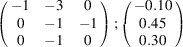	*a*′ = 10.4061 Å	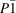
		*b* = 58.7985 Å		*b*′ = 10.5783 Å	
		*c* = 10.5783 Å		*c*′ = 53.6667 Å	
		α = 90.91°	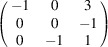	α′ = 90.80°	
		β = 110.17°		β′ = 94.55°	
		γ = 66.64°		γ′ = 110.17°	
		*V* = 5522.33 Å^3^		*V*′ = 5522.43 Å^3^	
					
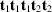	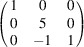	*a* = 10.4061 Å	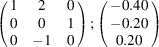	*a*′ = 10.4061 Å	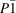
		*b* = 59.1192 Å		*b*′ = 10.5783 Å	
		*c* = 10.5783 Å		*c*′ = 53.6845 Å	
		α = 96.04°	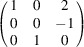	α′ = 91.02°	
		β = 110.17°		β′ = 94.62°	
		γ = 64.83°		γ′ = 110.17°	
		*V* = 5522.33 Å^3^		*V*′ = 5522.55 Å^3^	
					
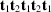	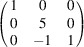	*a* = 10.4061 Å	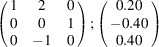	*a*′ = 10.4061 Å	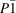
		*b* = 59.1192 Å		*b*′ = 10.5783 Å	
		*c* = 10.5783 Å		*c*′ = 53.6845 Å	
		α = 96.04°	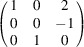	α′ = 91.02°	
		β = 110.17°		β′ = 94.62°	
		γ = 64.83°		γ′ = 110.17°	
		*V* = 5522.33 Å^3^		*V*′ = 5522.55 Å^3^	
					
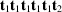	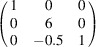	*a* = 10.4061 Å	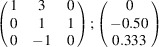	*a*′ = 10.4061 Å	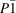
		*b* = 70.5494 Å		*b*′ = 10.5783 Å	
		*c* = 10.5783 Å		*c*′ = 64.1352 Å	
		α = 90.05°	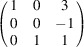	α′ = 90.11°	
		β = 110.17°		β′ = 90.04°	
		γ = 66.96°		γ′ = 110.17°	
		*V* = 6626.80 Å^3^		*V*′ = 6626.95 Å^3^	
					
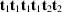	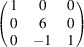	*a* = 10.4061 Å	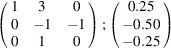	*a*′ = 10.4061 Å	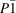
		*b* = 70.7518 Å		*b*′ = 10.5783 Å	
		*c* = 10.5783 Å		*c*′ = 64.3379 Å	
		α = 94.33°	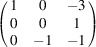	α′ = 85.40°	
		β = 110.17°		β′ = 88.33°	
		γ = 65.41°		γ′ = 69.83°	
		*V* = 6626.80 Å^3^		*V*′ = 6626.52 Å^3^	
					
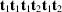	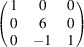	*a* = 10.4061 Å	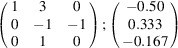	*a*′ = 10.4061 Å	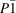
		*b* = 70.7518 Å		*b*′ = 10.5783 Å	
		*c* = 10.5783 Å		*c*′ = 64.3379 Å	
		α = 94.33°	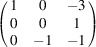	α′ = 85.40°	
		β = 110.17°		β′ = 88.33°	
		γ = 65.41°		γ′ = 69.83°	
		*V* = 6626.80 Å^3^		*V*′ = 6626.52 Å^3^	
					
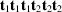	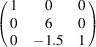	*a* = 10.4061 Å	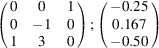	*a*′ = 10.5783 Å	*P*2_1_/*c*
		*b* = 71.3468 Å		*b*′ = 64.1316 Å	
		*c* = 10.5783 Å		*c*′ = 10.4061 Å	
		α = 98.57°	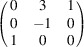	α′ = 90.04°	
		β = 110.17°		β′ = 110.17°	
		γ = 64.01°		γ′ = 90.12°	
		*V* = 6626.80 Å^3^		*V*′ = 6626.57 Å^3^	
					
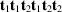	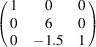	*a* = 10.4061 Å	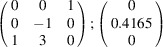	*a*′ = 10.5783 Å	*Pc*
		*b* = 71.3468 Å		*b*′ = 64.1316 Å	
		*c* = 10.5783 Å		*c*′ = 10.4061 Å	
		α = 98.57°	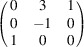	α′ = 90.04°	
		β = 110.17°		β′ = 110.17°	
		γ = 64.01°		γ′ = 90.12°	
		*V* = 6626.80 Å^3^		*V*′ = 6626.57 Å^3^	

**Table 4:** Sets of three diffraction peaks that may be used for identification of the individual polytypes of SFCA when there is more than one stacking sequence for a given value of *m* 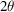
 values refer to Co *K*α_1_ radiation.

Layers in the sequence *m* and space-group symmetry	Stacking sequences	Sets of three peak positions (°2θ)
4 ( 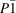 )	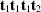	13.35°, 14.69°, 16.88°
4 (*P*2_1_/*c*)	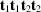	14.49°, 18.12°, 20.81°
5 ( 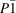 )	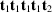	14.88°, 15.48°, 16.64°
5 ( 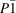 )	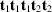	12.89°, 14.49°, 15.73°
5 ( 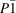 )	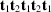	12.73°, 14.12°, 17.62°
6 ( 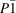 )	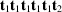	13.72°, 15.02°, 16.49°
6 ( 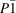 )	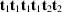	14.49°, 15.73°, 20.81°
6 ( 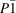 )	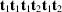	13.00°, 14.37°, 17.29°
6 (*P*2_1_/*c*)	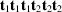	12.19°, 12.81°, 19.88°
6 (*Pc*)	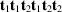	17.05°, 18.12°, 23.70°

**Table 5:** Sets of three diffraction peaks that may be used for identification of the individual polytypes of SFCA-I when there is more than one stacking sequence for a given value of *m* 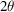
 values refer to Co *K*α_1_ radiation. Peaks involving contributions from family reflections are marked with an asterisk.

Layers in the sequence *m* and space-group symmetry	Stacking sequences	Sets of three peak positions (°2θ)
4 ( 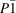 )	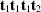	17.84°, 19.72°, 21.71°
4 (*P*2_1_/*c*)	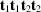	16.96°, 20.69°, 22.73°
5 ( 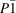 )	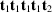	17.66°, 19.92°, 21.51°
5 ( 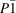 )	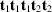	16.96°, 20.70°, 24.83°
5 ( 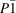 )	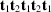	18.38°, 19.15°, 22.32°
6 ( 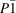 )	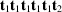	17.55°, 20.05°, *21.36°
6 ( 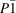 )	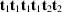	16.97°, 20.71°, 24.84°
6 ( 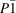 )	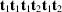	19.40°, *22.03°, *27.75°
6 (*P*2_1_/*c*)	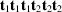	14.38°, 24.13°, 28.51°
6 (*Pc*)	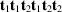	15.34°, 18.76°, 22.73°

## Data Availability

Two ZIP files containing the structural information of the ordered polytypes for SFCA and SFCA-I in CIF format, used for the simulation of the powder X-ray diffraction patterns, have been provided as supporting information.
